# Dimethyl fumarate attenuates experimental autoimmune neuritis through the nuclear factor erythroid-derived 2-related factor 2/hemoxygenase-1 pathway by altering the balance of M1/M2 macrophages

**DOI:** 10.1186/s12974-016-0559-x

**Published:** 2016-05-03

**Authors:** Ranran Han, Jinting Xiao, Hui Zhai, Junwei Hao

**Affiliations:** Department of Neurology, Tianjin Neurological Institute, Tianjin Medical University General Hospital, Anshan Road, Heping District, Tianjin, 300052 China

**Keywords:** Experimental autoimmune neuritis, Macrophages, Dimethyl fumarate, Guillain–Barré syndrome, Cytokine

## Abstract

**Background:**

Guillain–Barré syndrome (GBS) is an acute, post-infectious, immune-mediated, demyelinating disease of peripheral nerves and nerve roots. Dimethyl fumarate (DMF), a fumaric acid ester, exhibits various biological activities, including multiple immunomodulatory and neuroprotective effects. However, the potential mechanism underlying the effect of DMF in GBS animal model experimental autoimmune neuritis (EAN) is unclear.

**Methods:**

Using EAN, an established GBS model, we investigated the effect of DMF by assessing clinical score, histological staining and electrophysiological studies. Then, we further explored the potential mechanism by Western blot analysis, flow cytometry, fluorescence immunohistochemistry, PCR, and ELISA analysis. The Mann–Whitney *U* test was used to compare differences between control group and treatment groups where appropriate.

**Results:**

DMF treatment reduced the neurological deficits by ameliorating inflammatory cell infiltration and demyelination of sciatic nerves. In addition, DMF treatment decreased the level of pro-inflammatory M1 macrophages while increasing the number of anti-inflammatory M2 macrophages in the spleens and sciatic nerves of EAN rats. In RAW 264.7, a shift in macrophage polarization from M1 to M2 phenotype was demonstrated to be depended on DMF application. In sciatic nerves, DMF treatment elevated the level of the antioxidant transcription factor nuclear factor erythroid-derived 2-related factor 2 (Nrf2) and its target gene hemoxygenase-1 (HO-1) which could facilitate macrophage polarization toward M2 type. Moreover, DMF improved the inflammatory milieu in spleens of EAN rats, characterized by downregulation of messenger RNA (mRNA) of IFN-γ, TNF-α, IL-6, and IL-17 and upregulation of mRNA level of IL-4 and IL-10.

**Conclusions:**

Taken together, our data demonstrate that DMF can effectively suppress EAN, and the mechanism involves altering the balance of M1/M2 macrophages and attenuating inflammation.

**Electronic supplementary material:**

The online version of this article (doi:10.1186/s12974-016-0559-x) contains supplementary material, which is available to authorized users.

## Background

Guillain–Barré syndrome (GBS), known as an acute inflammatory disorder in the peripheral nervous system (PNS), can cause rapidly progressive, symmetrical weakness of the extremities [1]. Proven effective immunotherapies for GBS are plasma exchange and intravenous treatment with immunoglobulin G [1]. Of note, approximately 5 % of GBS patients die and up to 20 % suffer from severe disability, irrespective of whether they receive immunotherapies [2]. Hence, further researches are imperative to improve this situation.

Experimental autoimmune neuritis (EAN), a well-known animal model of GBS, effectively mimics clinical, histopathological, and electrophysiological features of GBS. EAN is characterized pathologically by a breakdown of the blood-nerve barrier, robust accumulation of reactive T cells and macrophages into the PNS, and demyelination of peripheral nerves [3]. Pro-inflammatory cytokines including IL-6, IFN-γ, IL-17, and TNF-α, mostly secreted by Th1 and Th17 cells, predominate in sciatic nerves and lymphoid organs during the acute phase of GBS and contribute to inflammatory impairment of peripheral nerves, whereas anti-inflammatory cytokines such as IL-4 and IL-10, mainly secreted by Th2 cells, are proven beneficial for the protection of inflammatory impairment of peripheral nerves [4, 5].

The polarized macrophages are generally termed pro-inflammatory M1 or classically activated macrophages and anti-inflammatory M2 or alternatively activated macrophages [6, 7]. M1 macrophages, which arise mainly in response to IFN-γ [8], are involved in the pathogenesis and course of EAN through their antibody-dependent cellular cytotoxicity (ADCC) and phagocytosis and myelin sheath damage [9]. M2 phenotype macrophages which arise mainly in response to IL-4 [8], however, contribute to recovery by promoting T-cell apoptosis and the secretion of anti-inflammatory cytokines [10], promoting myelin repair and axonal regeneration [11]. Evidence shows that a switch in macrophage phenotype from M1 to M2 could influence the pathogenesis of autoimmune and inflammatory diseases [12]. We hypothesize that treatment facilitating the switch of macrophage phenotype from M1 to M2 may favor the outcome of EAN.

Dimethyl fumarate (DMF), an orally bioavailable fumaric acid ester, has been used successfully as a drug for treating the autoimmune disorders such as psoriasis since the 1990s [13] and relapsing/remitting multiple sclerosis (MS) since 2013 [14]. Recent report shows that it can ameliorate EAN as well [15], but the exact mechanisms underlying its effect is still unclear. In addition, previous studies demonstrate that DMF ameliorates MS by reducing inflammation [16, 17] and enhancing activation of the nuclear factor erythroid-derived 2-related factor 2 (Nrf2) antioxidant pathway [18]. As a basic leucine-zipper (bZip) transcription factor, Nrf2 protects a variety of tissues and cells against oxidative, inflammatory, and electrophilic stress through antioxidant response element (ARE)-mediated induction of diverse phase II detoxification and antioxidant enzymes, including NAD(P)H quinone oxidoreductase 1 (NQO1) and hemoxygenase-1 (HO-1) [19, 20]. As the major anti-inflammatory and anti-oxidative enzyme that is regulated by activating Nrf2 [21], HO-1 induction has been reported to affect macrophage polarization toward M2 phenotype in experimental animal models of diabetes, Crohn’s disease, hypertension, alcoholic liver disease, and intestinal injury [22]. Herein, we hypothesized that HO-1 induction by DMF may exert a protection against EAN.

The objective of this study was to assess the effect of DMF in treating EAN and the underlying mechanisms. DMF was applied in a preventative and a therapeutic paradigm, and the effect on clinical, histological, and immunological parameters was assessed. It is demonstrated that DMF is effective in treating EAN and the mechanism of action involves mobilization of anti-inflammatory and immunomodulatory responses mediated by the Nrf2/HO-1 pathway.

## Methods

### Experimental animals and group assignments

Male Lewis rats, 6–8 weeks old (160–190 g), were purchased from the Vital River Corporation (Beijing, China). All animals were acclimated to the vivarium environment and were maintained under temperature-controlled conditions and a 12-h light/dark cycle for 1 week. Food and water were provided ad libitum. Animals were randomly assigned to three groups (preventative, therapeutic, or control, *n* = 6 per group) and each group was repeated 3 times. All efforts were made to minimize the number of animals used and any suffering they might experience. The experimental procedures were approved by the Animal Ethics Committee of the Tianjin Medical University.

### Induction and clinical evaluation of EAN

EAN was induced by injecting both hind footpads with 300 μl of an inoculum containing 300 μg of dissolved P0 peptide 180-199 (10 mg/ml; Bio-Synthesis Corporation). The peptide was dissolved in phosphate-buffered saline (PBS) (2 mg/ml) and then emulsified with an equal volume of complete Freund’s adjuvant (CFA; Difco) containing *Mycobacterium tuberculosis* (strain H37RA) to a final concentration of 1 mg/ml. Following immunization, clinical signs of EAN were scored and quantified as follows: 0 = normal; 1 = reduced tonus of the tail; 2 = limp tail; 3 = absent righting reflex; 4 = gait ataxia; 5 = mild paresis of the hind limbs; 6 = moderate paraparesis; 7 = severe paraparesis or paraplegia of the hind limbs; 8 = tetraparesis; 9 = moribund; and 10 = death.

### DMF treatment

Dimethyl fumarate (DMF; 97 % pure) (Sigma-Aldrich) was freshly prepared in 0.08 % carboxymethyl cellulose (CMC)/PBS. For preventative treatment, DMF solution was administered by oral gavage (25 mg/kg body weight/day) from day 1 to day 16 post-immunization (i.e., peak phase) or day 27 post-immunization (i.e., remission phase). For therapeutic treatment, DMF solution was administered by oral gavage at the same dose daily from the day on which the first clinical signs were observed, namely from day 7 to day 16 or 27 post-immunization. Control animals received the same volume of the vehicle solution (i.e., 0.08 % CMC/PBS). The dose of DMF was based on previously published studies [23, 24] and on preliminary studies done in our laboratory.

### Electrophysiological studies

Electromyographic (EMG) recordings of the left sciatic nerve were made on day 16 post-immunization (i.e., peak phase) using a fully digital Keypoint Compact EMG/NCS/EP recording system (Dantec Co). A single blind trial method was used to record evoked compound muscle action potential (CMAP) amplitudes and latencies of sciatic nerves, as previously described [25]. Rats were anesthetized first with chloral hydrate (intraperitoneally, 3 mg/kg). Two pairs of monopolar needle electrodes were used to stimulate the sciatic nerve and record the signals, respectively. After exposing the left sciatic nerve from the hip (proximal) to the ankle (distal), one pair of needle electrodes was inserted at the sciatic notch (hip/proximal) or the ankle (ankle/distal). The nerve stimulation parameters used to elicit CMAPs were 1-Hz pulses, with each pulse being 5 mA in amplitude and 0.3 ms in duration. The recording electrodes were positioned in the “belly” part of the gastrocnemius muscle to record evoked potentials from stimulating the sciatic nerve. The motor nerve conduction velocity (MNCV) was calculated by measuring the distance between stimulating cathode electrodes and then measuring the latency difference. The amplitude was calculated from the baseline to the maximal peak under the resulting CMAP curves. After electrophysiologic measurements were completed, the incision was sutured under an aseptic environment. Body temperatures of rats during electrophysiologic measurements were maintained above 34 °C by positioning a heating pad under the rat. For each animal, triplicate measurements were made.

### Histopathological assessment

Following nerve conduction studies, the sciatic nerves of each rat were harvested at the peak of disease (day 16 post-immunization). Six rats from each group received general anesthesia and were then perfused intracardially, first with 4 °C PBS for 2 min, followed by 4 % paraformaldehyde dissolved in PBS for 5 min. The sciatic nerves were rapidly removed and fixed in 4 % paraformaldehyde overnight at 4 °C, and then the nerves were embedded in paraffin.

To evaluate the extent of mononuclear cell (MNC) infiltration and demyelination, serial transverse sections (6 μm) were stained with hematoxylin-eosin (H&E) (Solarbio Science & Technology) and luxol fast blue (LFB), which contains 0.1 % LFB solution, 0.1 % Cresyl Echt Violet solution, and 0.05 % lithium carbonate solution. Infiltrating inflammatory cells in H&E-stained tissues were counted by image analysis using a Nikon Coolscope digital microscope (Nikon). Cell numbers were calculated per square millimeter from five random microscopic fields (200× magnification). All counts were performed in a blinded fashion. The average results were expressed as cells per square millimeter of tissue section. To evaluate the severity of demyelination, histological scores were calculated. Sections containing all perivascular areas were assessed by two independent observers (who were blinded to the treatment) according to the following semi-quantitative pathological/histological scale: 0, normal perivascular area; 1, mild cellular infiltrate adjacent to the vessel; 2, cellular infiltration plus demyelination in immediate proximity to the vessel; 3, cellular infiltration and demyelination throughout the section.

### Fluorescence immunohistochemistry

Following nerve conduction studies, the sciatic nerves were harvested at the peak of disease (i.e., day 16 post-immunization) as described above and post-fixed in 4 % paraformaldehyde overnight. The nerves were dehydrated sequentially in 15 % and then 30 % sucrose until the tissue equilibrated. After that, nerves were embedded in Tissue-tek medium (SAKURA) and snap-frozen in liquid nitrogen to expose antigenic sites for staining. Transverse sections (8 μm) were made on a cryostat (Leica Microsystems LM3050S) and then mounted on poly-l-lysine-coated slides and stored at −80 °C.

Fluorescence immunohistochemistry of the frozen sections was performed using standard protocols provided by the antibody manufacturers. Briefly, after bringing the slides to room temperature for 20 min, the mounted tissue was fixed in 4 % paraformaldehyde for 10 min and permeabilized in 0.3 % Triton X100 for 5 min. Sections were washed in cold PBS after each step. After blocking with 3 % BSA for 30 min at 37 °C, slides were incubated with primary antibodies: rabbit anti-Nrf2 (1:200, Abcam); rabbit anti-HO-1 (1:200, Abcam); mouse anti-CD68 (1:200, Abcam); goat anti-Iba1 (1:500, Abcam); rabbit anti-Iba1 (1:500, Wako); rabbit anti-iNOS (1:200, Abcam); rabbit-CD86 (1:200, Abcam); goat anti-Arginase-1 (1:200, Santa Cruz Biotechnology, Inc); and goat anti-CD206 (1:200, Santa Cruz Biotechnology, Inc) at 4 °C overnight. The next day, they were washed in PBS and then incubated for 60 min at room temperature with the following species-appropriate fluorochrome-conjugated secondary antibodies: Rhodamine (TRITC) AffiniPure donkey anti-goat IgG (H + L) (1:100, Jackson Immunoresearch); Rhodamine (TRITC)-conjugated AffiniPure goat anti-rabbit IgG (H + L) (1:100, Jackson, Immunoresearch); Rhodamine (TRITC)-conjugated AffiniPure goat anti-mouse IgG (H + L) (1:100, Jackson, Immunoresearch); Alexa Fluor® 488 conjugated donkey anti-rabbit IgG (H + L) (1:1000, Thermo Fisher Scientific); and Alexa Fluor® 488 conjugated donkey anti-goat IgG (H + L) (1:1000, Thermo Fisher Scientific). Finally, the slides were washed in PBS, and the tissue was coverslipped with fluoro-shield mounting medium containing DAPI (Abcam) to counterstain cell nuclei. Image analysis of stained cells of the entire area of each tissue section per sciatic nerve was performed using a Nikon Coolscope (Nikon). Positive cell numbers were calculated per square millimeter from three random microscopic fields (200× magnification). All counts were performed in a blinded fashion.

### Flow cytometry

The polarization state of macrophages derived from rat spleens was determined at the peak of disease (i.e., day 16 post-immunization) by flow cytometric analysis. In brief, spleens were removed under aseptic conditions and splenocytes were harvested. For extracellular staining, 1 × 10^6^ spleen MNCs were resuspended in 100 μl PBS/1 % BSA and were co-stained for cell surface CD11b conjugated with phycoerythrin (1:100, Abcam), Ly6G conjugated with FITC (1:100, Abcam) and/or CD206 (1:100, Abcam) for 45 min at room temperature (RT) following manufacturer’s specification. For intracellular staining, cells were first stained with CD11b and Ly6G as above, then fixed with Fixation Buffer (BioLegend), permeabilized with Permeabilization Wash Buffer (BioLegend), and then stained with a primary antibody for intracellular iNOS (1:100, Abcam) and Alexa Fluor®647-labelled donkey anti-rabbit IgG (H&L) secondary antibody (1:100, Abcam). MNCs were gated by forward and sideward scatter. Surface and intracellular molecule expression were assessed by determining the positive cell percentage. Cells from all groups were collected and analyzed at each time point on the same day with the same cytometer settings. Flow cytometric data were acquired using a FACSAria™ flow cytometer (BD Biosciences) and analyzed with FlowJo software version 7.6.1 (flowjo.com).

### Western blot analysis

After removal, part of the sciatic nerves were immediately stored in liquid nitrogen until needed for protein isolation by TRIzol (Ambion). All procedures were performed following manufacturer’s introduction for obtaining total protein. After boiling, samples were adjusted to equal content and volume before being loaded on 10 % SDS-PAGE gels and electrophoretically separated. Proteins were then transferred to PVDF membranes (Millipore) followed by blocking. Proteins were visualized by using primary antibodies for Nrf2 (1:1000, Abcam) and HO-1 (1:2000, Abcam), actin (1:1000, Zhongshanjinqiao), and then by incubating with goat anti-rabbit horseradish peroxidase-conjugated secondary antibody (1:5000, TRANSGENE BIOTECH) and goat anti-mouse secondary antibody (1:5000, TRANSGENE BIOTECH). The protein-specific signals were detected using a Bio-Rad 721BR08844 Gel Doc Imager (Bio-Rad).

### Lymphocyte proliferation assay

Antigen-specific lymphocyte proliferation was measured by MTS (3-(4, 5-dimethylthiazol-2-yl)-5-(3-carboxymethoxyphenyl)-2-(4-sulfophenyl)-2H-tetrazolium) assays on day 16 post-immunization as described previously [26]. Briefly, spleens were removed under aseptic conditions and splenocytes were harvested. Single-cell suspensions of MNCs at a density of 2 × 10^6^ cells/ml were allocated with 100-μl culture medium into 96-well microtiter plates. The culture medium was RPMI1640 (containing 2.05 mM glutamine; HyClone) supplemented with 1 % (*v*/*v*) HEPES buffer solution (Gibco), 0.1 % (*v*/*v*) 2-mercaptoethanol (Gibco), 1 % (*v/v*) sodium pyruvate (Gibco), and 1 % (*v*/*v*) Pen Strep (Gibco), and 10 % (*v*/*v*) fetal bovine serum (FBS; Gibco). MNCs were cultured in the presence of either P0 peptide 180–199 (10 μg/ml; Bio-Synthesis Corporation) or without antigen. The concentration of the peptide used was based on previous studies [26, 27]. Following incubation for 72 h at 37 °C and 5 % CO_2_, MTS solution (Promega) was added and cells were incubated for an additional 4 h. The absorbance was measured at 490 nm using a microtiter plate reader (Titertek). Since we administered the drug in vivo, we could not calculate an inhibitory rate. The data are presented as mean OD ± SEM. The experiment was performed with MNCs from four different rats in each group, and MNCs from each rat were done in triplicate.

### Real-time quantitative polymerase chain reaction (RT-PCR)

Spleens were removed under aseptic conditions and splenocytes were harvested as described above. 10^7^ splenic cells were stored for proceduring RNA isolation by TRIzol (Ambion). All actions were performanced following instruction book to get total RNA (10 μg/sample). Total RNA was reverse transcribed into complementary DNA (cDNA) using TransScript First-Strand cDNA Synthesis SuperMix (TRANSGEN BIOTECH). All the procedures were strictly performed as per instructions. The primers used to measure gene expression are the following: IFN-γ (sense, TCGCACCTGATCACTAACTTCTTC; antisense, CGACTCCTTTTCCGCTTCC), TNF-α (sense, TGA TCG GTC CCA ACA AGG A; antisense, TGC TTG GTG GTT TGC TAC GA), IL-4 (sense, TGA TGG GTC TCA GCC CCC ACC TTG C; antisense, CTT TCA GTG TTG TGA GCG TGG ACT C), IL-17 (sense, TGGACTCTGAGCCGCATTGA; antisense, GACGCATGGCGGACAATAGA),IL-4 (sense, TGATGGGTCTCAGCCCCCACCTTGC; antisense, CTTTCAGTGTTGTGAGCGTGGACTC), β-actin (sense, CCGTCTTCCCCTCCATCGT; antisense, ATCGTCCCAGTTGGTTACAATGC). The PCR program was run at 95 °C for 10 min then 40 cycles at 95 °C for 15 s and 60 °C for 1 min. The results were automatically analyzed by the ABI Stepone Plus instrument, and the method of 2^–△△Ct^ (△Ct represents the difference of threshold cycle value between the target gene and the inner control; △△Ct represents the difference of △Ct between different groups) was used to analyze the messenger RNAs (mRNA) expression. Results were calculated as levels of target mRNAs relative to β-actin (four samples from each group were analyzed by PCR).

### ELISA for cytokine profile

The supernatants of splenic MNCs (2 × 10^6^ cells/ml) cultured with P0 peptide 180-199 (10 μg/ml)were collected after incubation for 72 h at 37 °C and 5 % CO_2_. Simultaneous quantitative analysis of six cytokines, including IL-1α, IL-4, IL-6, IL-10, IFN-γ, TNF-α, was performed using a multi-analyte ELISArray Kit (QIAGEN) according to the manufacturer’s instructions. Determinations were performed in triple, and results are expressed as mean OD ± SEM (*n* = 4).

### In vitro cell culture

To further investigate the effects of DMF on macrophage polarization, we used immortalized murine macrophage cell line RAW 264.7 in vitro. RAW 264.7 cells were grown in complete RPMI 1640 medium (Life Technologies) containing penicillin (100 U/ml), streptomycin (100 U/ml), and 10 % FBS (Gibco), maintained at 37 °C and 5 % CO_2_. Briefly, 2 × 10^5^ cells were seeded into 24-well cell culture plates, triplicated either on coverslips pretreated with polylysine or not, and cultured for 24 h. Then, cells were stimulated with LPS (Sigma-Aldrich) for 24 h, and afterwards DMF (0, 20, 50, 100 μM) was added and incubated for another 24 h. Thereafter, cells on slides were prepared for immunocytochemistry staining and the other cultured cells were harvested and centrifuged for RNA analyses (see below). The staining procedure of cells was similar to that of the tissue slides as mentioned above. Immunocytochemistry of macrophages on the slides used the following primary antibodies. To detect M1 macrophage markers, we used rabbit antibody to iNOS (1:200, Abcam). To detect M2 markers, we used goat antibody to Arginase-1 (1:200, Santa Cruz Biotechnology, Inc). Antibodies used to mark pan macrophage markers included mouse antibody to goat anti-Iba1 (1:500, Abcam); rabbit anti-Iba1 (1:500, Wako). Species-appropriate fluorochrome-conjugated secondary antibodies were added. Cells were observed by using fluorescence microscopy (Nikon). Five different fields of each slide were acquired; the pictures of each group were collected from triple-cultured cells. Fluorescence intensity of iNOS and Arginase-1 (Arg1) was analyzed semi-quantitatively by image analysis software (Image ProPlus 6.0 Software). Results were provided as relative intensity comparing DMF-treated cells to PBS-treated cells.

Total RNA from cultured cells was prepared as described above according to the manufacturer’s instructions. Total RNA was reverse transcribed into cDNA using TransScript First-Strand cDNA Synthesis SuperMix (TRANSGEN BIOTECH). The mRNA expression levels were measured by real-time PCR using the following primers: β-actin (sense, TGG AAT CCT GTG GCA TCC ATG AAA; antisense, TAA AAC GCA GCT CAG TAA CAG TCC G); TNF-α (sense, AAC TAG TGG TGC CAG CCG AT; antisense, CTT CAC AGA GCA ATG ACT CC); iNOS (sense, CAG CTG GGC TGT ACA AAC CTT; antisense, CAT TGG AAG TGA AGC GTT TCG); Arg 1 (sense, TTA GGC CAA GGT GCT TGC TGC C; antisense, TAC CAT GGC CCT GAG GAG GTT C); and IL-10 (sense, TCA TTC ATG GCC TTG TAG ACA C; antisense, AGC TGG ACA ACA TAC TGC TAA C). The PCR program was run at 95 °C for 10 min, then 40 cycles at 95 °C for 15 s, and finally at 60 °C for 1 min. The results were automatically analyzed by an ABI SteponePlus instrument, and the method of 2^–△△Ct^ was used to analyze mRNA expression. Results were calculated as levels of target mRNAs relative to those from untreated RAW 264.7 cells. Four samples from each group were analyzed by PCR.

### Evaluation and statistical analysis

The Mann–Whitney *U* test was used to compare differences between control group and treatment groups where appropriate (GraphPad Prism 5.0). Data are presented as means ± SEM. For all statistical analyses, the level of significance was set at *p* < 0.05.

## Results

### DMF treatment attenuates clinical severity, histological changes, and inflammatory cell accumulation in EAN

**Fig. 1:**
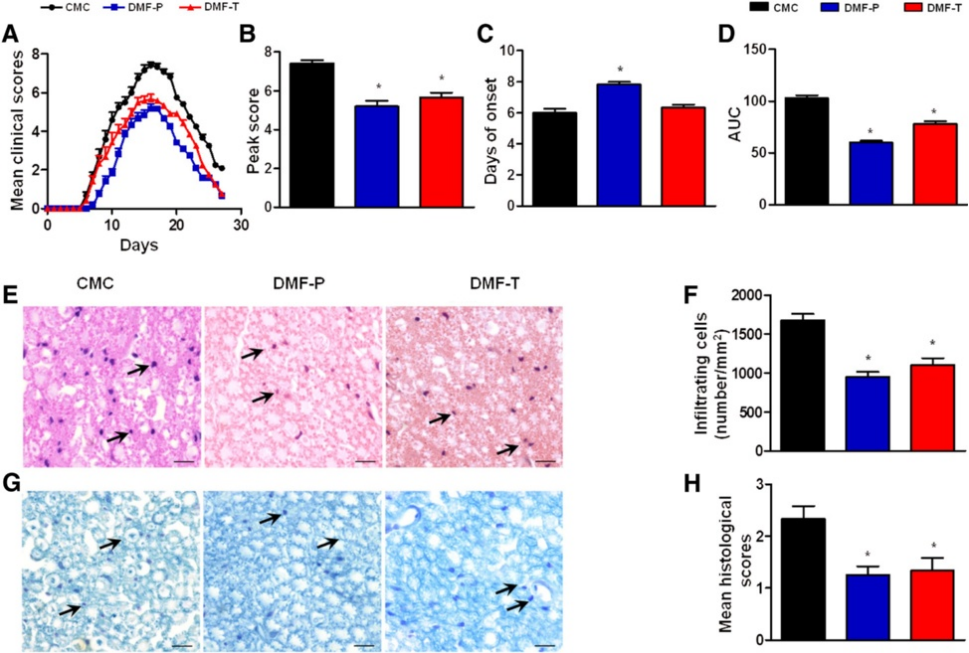
DMF treatment attenuates the severity of EAN. EAN was induced in rats by immunization on day 0 with P0 peptide 180-199 (300 μg) emulsified in complete Freund’s adjuvant and dissolved in vehicle. In a preventative treatment paradigm, DMF was administered from day 1 to day 27 (25 mg/kg, once daily). By contrast, in a therapeutic treatment paradigm, DMF was administered from day 7 to day 27 at the same 25 mg/kg dose, once daily. In a control condition, EAN rats received an equivalent volume of vehicle. Clinical scores (0–10, with 0 being normal to 10 being so severely impaired that the animals died) were obtained daily post-immunization. **a** EAN clinical scores were significantly better in DMF-treated groups, but the pattern of improvement over time was different. The rats in the preventative treatment group exhibited significantly better clinical scores from day 6 to day 27 compared with the rats in the CMC group (*p* < 0.05 on each time point). The rats in the therapeutic group had significantly better clinical scores from day 10 to day 27 compared with the rats in the CMC group (*p* < 0.05 on each time point). **b**–**d** DMF reduced the peak score, the day of disease onset, and the area under the curve (AUC) in EAN rats. On day 16 post-immunization, sciatic nerves of each group were harvested for H&E staining (**e**) and LFB staining (**g**) to assess changes in infiltration and demyelination. Representative photomicrographs are shown for each group. The mean number of inflammatory cells per square millimeter of tissue section was determined as described in the “Methods” section, and summaries are shown in **f**. Mean histological scores are shown in **h**. Scores were calculated as described in the “Methods” section. Comparisons between the CMC group and DMF-treated groups were performed using Mann–Whitney *U* tests. *Arrows* showed the inflammatory cells and the demyelination of sciatic nerves. Scale bar in **e** and **g** is 10 μm. The results are presented as means ± SEM (*p < 0.05 for comparison between CMC and DMF-treated groups, *n* = 6 per group). *CMC* vehicle control group, *DMF-P* preventative DMF group, *DMF-T* therapeutic DMF group. The experiment was repeated 3 times with similar results

Treatment of EAN in our rat model with DMF was investigated in two different paradigms (Fig. 1). In the preventative paradigm (blue line), we administered vehicle or DMF (25 mg/kg/day) by gavage after immunization. In the therapeutic paradigm (red line), the treatment was started when the first clinical symptoms occurred on day 7 post-immunization. The severity of EAN was reduced in both the preventative and therapeutic treatment groups compared to the control group (CMC), as measured by clinical severity scores. The rats in the preventative treatment group exhibited significantly better clinical scores from day 6 to day 27 compared with the rats in CMC group (*p* < 0.05 on each time point). By contrast, clinical improvement of rats in the therapeutic group was delayed, with their clinical scores being lower than that of the rats in CMC group from day 8 to day 27 and the difference was significant only from day 10 to day 27 (*p* < 0.05 on each time point). In addition, the mean peak clinical score was 5.20 ± 0.30, 5.67 ± 0.25, and 7.42 ± 0.15 in the preventative, therapeutic, and CMC groups, respectively (Fig. 1a, b). The preventative treatment group delayed the onset of the first clinical sign of impairment (Fig. 1a, c). The day of EAN onset was day 7.83 ± 0.17 in the preventative group, 6.33 ± 0.21 in the therapeutic group, and 6.00 ± 0.26 in the CMC group (Fig. 1c). DMF also reduced the AUC of the motor deficit assessed by clinical scoring (Fig. 1d). Overall, both preventative and therapeutic DMF treatment ameliorated EAN.

At the maximum of the clinical course of EAN (i.e., day 16 post-immunization), we evaluated the histopathology of the rats’ sciatic nerves. H&E staining was used to show inflammatory cell infiltration, and LFB staining was used to show myelin demyelination. Treating EAN rats with DMF in both treatment paradigms lowered the number of inflammatory cells (Fig. 1e, f). The incidence of demyelination and inflammatory cell infiltration was reduced by DMF treatment in both paradigms (Fig. 1g, h). When the stained tissue was semi-quantitatively graded, the mean histological scores were markedly higher in the CMC group (2.33 ± 0.25) compared to the scores of preventative (1.25 ± 0.17, *p* < 0.05) and therapeutic (1.33 ± 0.25, *p* < 0.05) groups (Fig. 1h).

### DMF protects against EAN-induced peripheral nerve injury

**Fig. 2:**
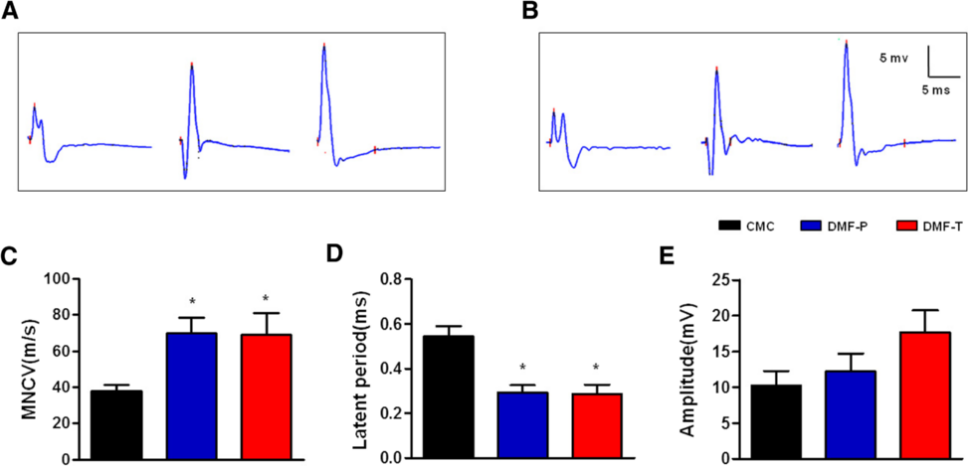
DMF protects against peripheral nerve conduction deficits in EAN rats. Representative electrophysiological recordings of motor nerve compound muscle action potentials (CMAPs) evoked from stimulation of the fibular head (**a**) or ankle regions (**b**) of the sciatic nerve are shown for CMC, DMF-P, and DMF-T rats, respectively, from *left* to *right*. **c** Compared to the CMC group, DMF-treated groups were protected from EAN-induced damages of mean motor nerve conduction velocity (MNCV). **d** Compared to the CMC group, DMF-treated groups also exhibited better distal motor latencies of CMAPs. **e** DMF treatment improved the amplitude of CMAPs when compared to CMC groups, while we observed a non-significant trend. Comparisons between the CMC group and DMF-treated groups were performed using Mann–Whitney *U* tests. Data shown are the means ± SEM (*p < 0.05 for comparison between CMC and DMF-treated groups; *n* = 5–6). *CMC* vehicle control group, *DMF-P* preventative DMF group, *DMF-T* therapeutic DMF group. The experiment was repeated 3 times with similar results

The ability of DMF to protect against EAN-induced peripheral nerve injury was assessed by evoked response electrophysiology at day 16 post-immunization in the sciatic nerve. It is well known that EAN rats exhibit decreasing CMAP amplitude and MNCV and lengthening of CMAP latency. In agreement with the clinical findings shown in Fig. 1, DMF prevented the development of peripheral nerve deficits in both DMF-treated groups (Fig. 2). For the MNCV, the CMC group’s mean conduction velocity was slower than the preventative and therapeutic groups’ conduction velocity (37.73 ± 3.49 vs. 70.08 ± 8.25 m/s and 69.24 ± 11.73 m/s, *p* < 0.05, Fig. 2c). For CMAP latency, the preventative group and therapeutic group also showed a beneficial effect of DMF on CMAP latency, which was shorter than the CMC group’s average latency (0.29 ± 0.03 and 0.29 ± 0.04 ms vs. 0.55 ± 0.04 ms, *p* < 0.05, Fig. 2d). Also, the CMAP mean amplitudes of the two DMF treatment groups were greater (12.25 ± 2.52 and 17.72 ± 3.03 mV, respectively, Fig. 2e) compared to the CMC group (10.25 ± 2.04 mV), but this was not significant.

### DMF promotes M2 macrophage polarization in EAN rats and in vitro

**Fig. 3:**
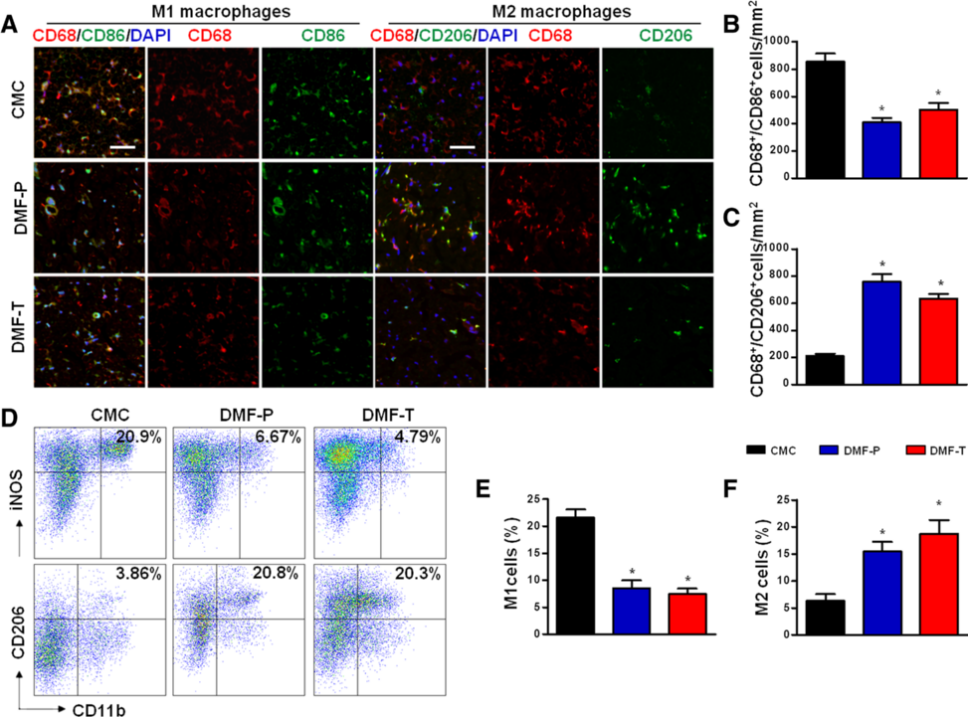
DMF promotes M2 macrophage polarization in EAN. DMF was administered orally to EAN rats (*n* = 6 in each group) from day 1 to day 16 or day 7 to day 16 post-immunization for preventative or therapeutic treatment, respectively. On day 16 post-immunization, sciatic nerves of each group of rats were harvested for fluorescence immunohistochemistry, and spleen mononuclear cells (MNCs) were isolated for flow cytometry, in both cases to analyze the proportion of M1 macrophages and M2 macrophages in EAN after DMF treatment paradigms. **a** Fluorescence photomicrographs showing M1 and M2 macrophages in the sciatic nerves of EAN rats. Tissue sections were immunofluorescence stained for markers of M1 and M2 macrophages as indicated. Scale bar is 50 μm. **b** Quantitation of immunohistochemistry. Counts per square millimeter of CD68^+^/CD86^+^ cells (M1 phenotype) in the sciatic nerve showed that DMF reduced the number of M1 macrophages in both DMF treatment paradigms. **c** Counts per square millimeter of CD68^+^/CD206^+^ cells (M2 phenotype) showed that DMF-treated groups increased the number of M2 macrophages in both DMF treatment paradigms compared to the CMC group. **d** Flow cytometry results of spleen MNCs showed the same pattern of M1/M2 phenotype shift as a result of both DMF-preventative and DMF-therapeutic treatment paradigms. **e** Quantitation of flow cytometry. Percentage of M1 phenotype in spleen MNCs was lower in both DMF-treated groups. **f** Percentage of M2 macrophages in spleen MNCs was greater in both DMF-treated groups. Comparisons to CMC group were performed using the Mann–Whitney *U* test; quantitation shows means ± SEM (*p < 0.05 for comparison between CMC and DMF-treated groups; *n* = 4–5). *CMC* control group receiving vehicle, *DMF-P* preventative DMF treatment, *DMF-T* therapeutic DMF treatment. The experiment was repeated 3 times with similar results

To further investigate the mechanism of DMF’s beneficial effect in EAN, we assessed possible DMF-related changes in macrophage polarization in sciatic nerves and in spleens of EAN rats. Double-immunohistochemical staining was used to identify the proportion of M1 and M2 cells in sciatic nerves. Flow cytometry was also used to investigate polarization state of MNCs derived from spleens of EAN rats treated in the different DMF paradigms. In sciatic nerves harvested on day 16 post-immunization, DMF-preventative and DMF-therapeutic groups both displayed elevated local expression of M2 macrophages and reduced expression of M1 macrophages compared to the CMC group (Fig. 3a–c, *p* < 0.05 and Additional file 1: Figure S1). Flow cytometry results of spleen MNCs followed the same group patterns of M1/M2 expression. Both the DMF-preventative and DMF-therapeutic groups showed elevated expression of M2 and reduced expression of M1 macrophages in EAN spleens compared to the CMC group (Fig. 3d–f, *p* < 0.05).

**Fig. 4:**
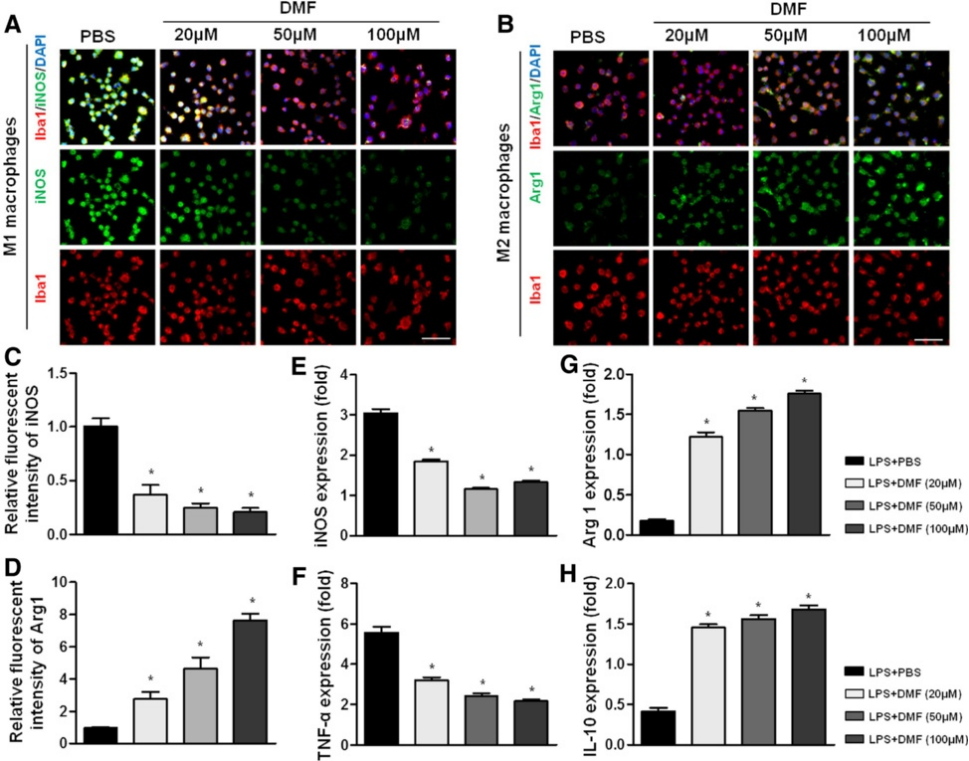
DMF applied in vitro induces M2 macrophage polarization. The murine macrophage cell line RAW 264.7 was used to verify DMF’s effect on phenotype shift. M1 macrophage phenotype was induced first by LPS (5 μg/ml) for 24 h, and then DMF (20, 50, or 100 μM) or vehicle was applied to the culture and allowed to incubate for 24 h. In vitro application of DMF reduced the immunocytochemical staining of iNOS^+^ cells (**a**) and increased the immunocytochemical staining of Arg1^+^ cells (**b**). The results were significant by semi-quantitative analysis of immunofluorescence for iNOS (**c**) and Arg1 (**d**). DMF application significantly attenuated mRNA expression of iNOS (**e**) and TNF-α (**f**). mRNA expression of Arg 1 (**g**) and IL-10 (**h**) increased significantly after DMF treatment. These results suggested a polarization shift from M1 to M2 phenotype. Scale bars in **a** and **b** are 50 μm. Comparisons between the DMF-treated groups and DMF-untreated control group (PBS) were performed using the Mann–Whitney *U* test. Results are expressed as means ± SEM (*p < 0.05 compared to the group stimulated with LPS only, *n* = 4)

As a third line of evidence, we assessed the in vitro polarization shift of macrophages after DMF treatment using the murine macrophage cell line, RAW 264.7. The M1 macrophage phenotype was first induced by in vitro LPS application (5 μg/ml) for 24 h. Various concentrations of DMF (20, 50, and 100 μM) were then added to the culture, and the cells were incubated for another 24 h. As shown in Fig. 4, in vitro DMF application reduced the expression of iNOS and increased the expression of Arg1 significantly (Fig. 4a–d, *p* < 0.05) detected by fluorescence immunohistochemistry. DMF application also attenuated iNOS and TNF-α mRNA expression and increased Arg1 and IL-10 mRNA expression (Fig. 4e–h, *p* < 0.05). These results suggested a shift in polarization from the M1 to M2 phenotype. Therefore, we verified in vitro that DMF can promote a shift to M2 polarization, and together with our previous results, these results showed that the shift can possibly be induced in vivo as well by DMF treatment.

### DMF increases Nrf2 and HO-1 protein levels in sciatic nerves of EAN rats

**Fig. 5:**
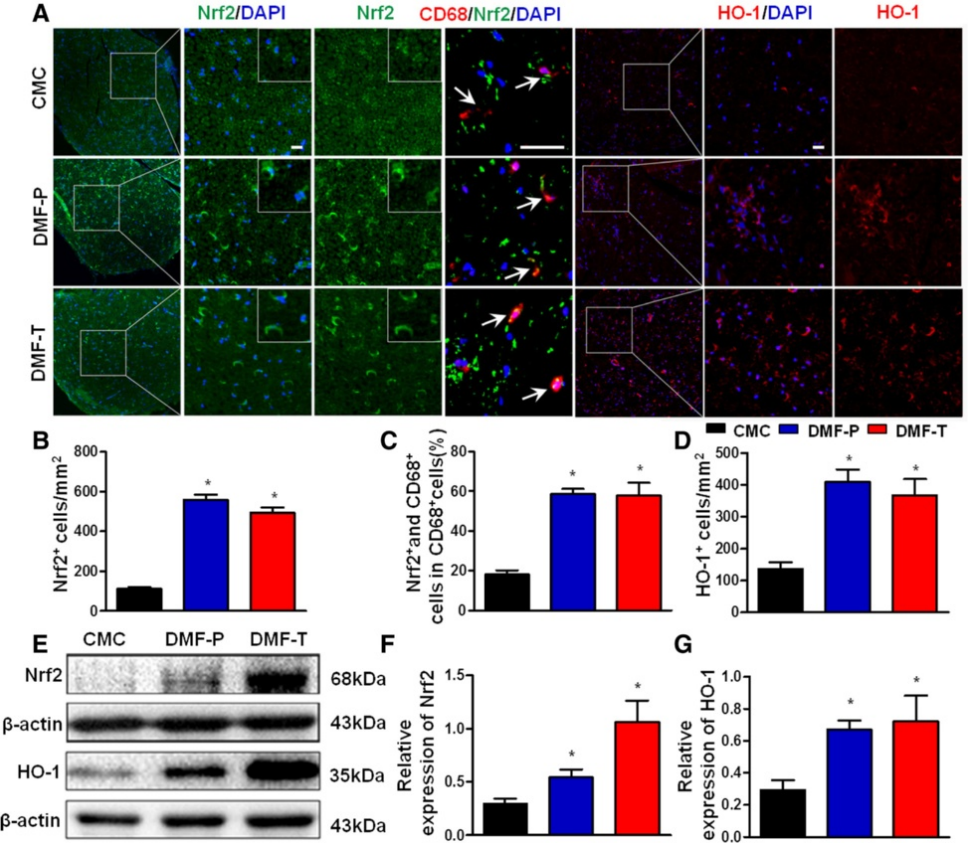
Preventative and therapeutic DMF treatment increases the production of Nrf2, the percentage of Nrf2 in macrophages, and the level of HO-1 in sciatic nerves of EAN rats. DMF was administered orally to EAN rats (*n* = 6 in each group) from day 1 to day 16 and day 7 to day 16 post-immunization for preventative and therapeutic treatment, respectively. On day 16 post-immunization, sciatic nerves of rats from each group were harvested for fluorescence immunohistochemistry and Western blotting. DAPI counterstaining was used in tissue sections for cellular identification. **a** Fluorescence photomicrographs of anti-Nrf2 (green), anti-CD68 (red), and anti-HO-1 (red) staining. In both the DMF-preventative and DMF-therapeutic groups, Nrf2 and HO-1 stainings were more intense and robust when compared to the CMC group. Co-staining macrophages with Nrf2 showed that DMF-treated groups had a higher radio of Nrf2 positive macrophages than CMC group. Scale bar is 20 μm. **b** Quantitation of cellular anti-Nrf2 staining. There were significantly more Nrf2^+^ cells per square millimeter in the DMF-treated groups compared to the CMC group. **c** Fluorescence photomicrographs and quantitation of anti-Nrf2 and anti-CD68 staining showed that in both the DMF-preventative and DMF-therapeutic groups, the percentage of Nrf2- and CD68-positive cells in CD68-positive cells was higher when compared to the CMC group. **d** Quantitation of cellular anti-HO-1 staining. There was significantly more HO-1^+^ cells in the DMF-treated groups compared to the CMC group. **e** Western blotting of Nrf2 and HO-1 protein compared to β-actin standard. DMF-preventative and DMF-therapeutic groups both showed increased expression of Nrf2 and HO-1 in sciatic nerves when compared to the CMC group. **f**, **g** Quantitation of Western blotting of Nrf2 and HO-1. Relative expression of Nrf2 and HO-1 in DMF-treated groups was significantly greater compared to the CMC group. Comparisons between the CMC group and DMF-treated groups were made by Mann–Whitney *U* test. Results are expressed as means ± SEM (*p < 0.05 compared to the CMC control group; *n* = 4–5). The experiment was repeated 3 times with similar results

Nrf2 protein regulates the expression of antioxidant proteins that protect against oxidative damage related to inflammation, and HO-1 is a key downstream mediator of Nrf2. As M2 macrophages highly express HO-1 [22], we explored the effect of DMF on the level of Nrf2 and HO-1. Sciatic nerves were taken from EAN rats on post-immunization day 16 for fluorescence immunohistochemistry and Western blotting analysis to study the cellular expression of Nrf2/HO-1. In sciatic nerve tissue sections, DMF-preventative and DMF-therapeutic groups showed robust localized cellular expression of Nrf2 and HO-1 compared to the CMC group (Fig. 5a, b, d, *p* < 0.05). Furthermore, detection by Western blotting also showed an increased level of Nrf2 and HO-1 (Fig. 5e–g, *p* < 0.05). In addition, co-staining macrophages with Nrf2 showed that DMF-treated groups had a higher radio of Nrf2-positive macrophages than CMC group (Fig. 5a, c, *p* < 0.05).

### DMF ameliorates lymphocyte proliferative responses and alters cytokine profile

**Fig. 6:**
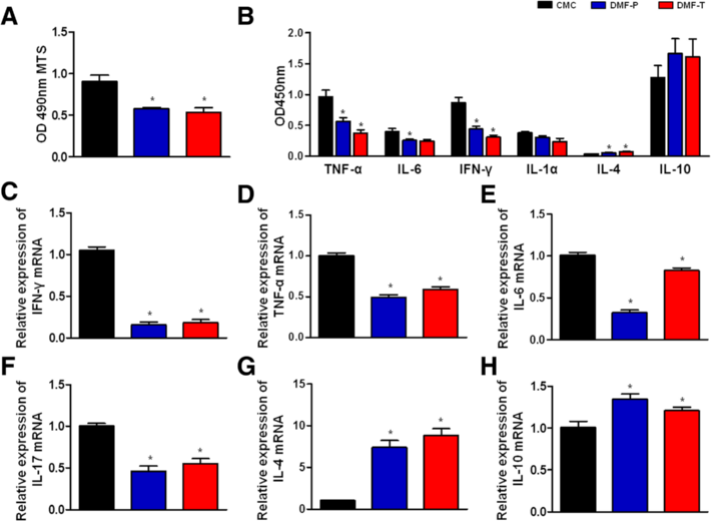
DMF ameliorates lymphocyte proliferative responses and alters cytokine profiles. Splenic mononuclear cells (MNCs) from each group (*n* = 6) were harvested on day 16 post-immunization. **a** MNCs proliferation following DMF treatment. After culturing MNCs for 72 h in the presence of P0 peptide 180-199 (10 μg/ml), MNC proliferation was analyzed by MTS assay. When compared to the CMC group, a significant reduction in proliferation was observed in DMF-preventative and DMF-therapeutic groups. **b** Cytokine profiles of the supernatants of spleen MNCs cultured with P0 peptide 180-199 (10 μg/ml) for 72 h, as measured by ELISA. The levels of TNF-α, IL-6, and IFN-γ decreased greatly, but the level of IL-4 increased compared to the CMC group. A trend in increasing IL-10 levels and decreasing IL-1α levels was evident but not statistically significant. **c**–**h** IL-6, IL-4, IL-10, IFN-γ, TNF-α, and IL-17 mRNA expression levels in the splenocytes of EAN rats, as measured by RT-PCR. The level of pro-inflammatory cytokines IFN-γ, TNF-α, IL-6, and IL-17 decreased in DMF-treated groups, while the level of anti-inflammatory cytokines IL-4 and IL-10 increased significantly. Each experiment was performed in triplicate for each group. Comparisons between the CMC group and DMF-treated groups were performed using Mann–Whitney *U* tests. Data shown are means ± SEM (*p < 0.05 for comparison between CMC and DMF-treated groups, *n* = 4). The experiment was repeated 3 times with similar results

MNCs prepared from EAN rat spleens on day 16 post-immunization were stimulated in vitro with P0 peptide 180-199 (10 μg/ml) or without peptide for 72 h to evaluate the effect of DMF on lymphocyte-proliferative responses (Fig. 6a). When compared to the CMC group, a significant reduction in lymphocyte proliferation was observed in the preventative group and therapeutic group (0.91 ± 0.08 vs. 0.58 ± 0.02, and 0.54 ± 0.05, respectively) (Fig. 6a, *p* < 0.05), as measured by MTS assay with P0 peptide stimulation. The same pattern of lymphocyte proliferation was observed without peptide stimulation, and there was no significant difference between DMF treatment groups and the control group (data not shown).

As is well documented, the expression profiles of cytokines affect the outcome of EAN. Therefore, we semi-quantitatively analyzed the effects of DMF treatment on the cytokine profiles produced in vitro by MNCs from EAN rats in response to the presence of P0 peptide 180-199 using multi-cytokine ELISA kits. As shown in Fig. 6b, the production of pro-inflammatory cytokines (IL-6, IFN-γ, IL-1α, and TNF-α) by MNCs cultured with P0 peptide 180-199 (10 μg/ml) was reduced in both DMF treatment groups compared to that in the CMC group. While TNF-α, IL-6, and IFN-γ were all significantly reduced (Fig. 6b, *p* < 0.05), the levels of IL-1α were not reliably different from those in the CMC group. By contrast, the levels of the anti-inflammatory cytokines IL-4 and IL-10 were upregulated. However, only levels of IL-4 were significantly greater compared to the CMC group (Fig. 6b, *p* < 0.05); large variability in IL-10 levels among the groups apparently reduced the statistical power to detect a reliable difference.

We confirmed these changes in cytokine production in DMF-treated MNCs by using RT-PCR of splenocyte samples. As shown in Fig. 6c–h, IFN-γ, TNF-α, IL-6, and IL-17 mRNA levels were greatly reduced in DMF-treated groups compared to those in the CMC group (Fig. 6c–f, *p* < 0.05), while IL-4 and IL-10 mRNA levels increased significantly (Fig. 6g, h, *p* < 0.05).

## Discussion

In the present study, DMF therapy suppressed the effects of EAN, manifested overtly in a delay of clinical symptom onset and in a reduction in the severity of paralysis. These beneficial overt effects were associated with reduced inflammation and demyelination of the sciatic nerves. We also observed a decrease in the often-damaging M1 macrophage phenotype and an increase in the M2 phenotype in both sciatic nerves and spleens of DMF-treated rats and in vitro. In addition, the level of Nrf2 and HO-1 increased significantly in DMF-treated groups. Furthermore, DMF improved the inflammatory environment by depressing the level of pro-inflammatory cytokines and increasing the level of anti-inflammatory cytokines.

Assessing neurological symptoms through clinical scores, we observed that DMF greatly improved EAN symptoms by reducing paralysis severity, delaying the onset of the first signs of EAN, decreasing the peak clinical score, and reducing motor deficits. EMG studies revealed that DMF generally protected nerves from EAN-induced peripheral nerve injury by improving MNCV and amplitudes of CMAPs and by reducing the distal motor latency compared to the CMC group. Histopathologically, EAN is characterized by inflammatory cell infiltration and nerve demyelination. In the present study, both preventative and therapeutic DMF treatment significantly suppressed the accumulation of inflammatory cell infiltration and demyelination when compared to the CMC group. Taken together, these results demonstrate that DMF had beneficial effects in EAN rats.

Macrophages are broadly divided into two groups: classically activated (M1) and alternatively activated (M2) macrophages [28]. M1-type macrophages release cytokines that inhibit the proliferation of surrounding cells and damage contiguous tissue, whereas M2-type macrophages release cytokines that promote the proliferation of contiguous cells and tissue repair [10]. Furthermore, alternatively activated M2 macrophages play an immunomodulatory role in EAN [29, 30], leading to a favorable outcome. Consistent with the previous findings, the present study showed that the ameliorated outcome of EAN rats was associated with the polarization of macrophages toward the M2 phenotype. There were many more M2-type and fewer M1-type macrophages in the sciatic nerves of DMF-treated EAN rats. Besides, flow cytometry results showed that splenic macrophages in DMF-treated EAN rats polarized toward the M2 type. This finding was also consistent with in vitro findings showing that DMF application in the culture bath directly affected macrophages differentiation. Indeed, treating LPS-stimulated macrophages with DMF significantly reduced mRNA expression of inflammatory molecules, such as iNOS and TNF-α, and induced mRNA expression of anti-inflammatory cytokines, such as IL-10. These data supported the idea that DMF improved the outcome of EAN by promoting M2 type differentiation.

Based on previous mechanistic studies with cell culture models and MS, it was proposed that DMF’s therapeutic mechanism is mainly the activation of Nrf2 [19]. Evidences show that Nrf2 is beneficial for other autoimmune and inflammatory diseases as well [31–35]. Corroborating these studies, our study revealed that in EAN, DMF upregulated Nrf2 in vivo. It is reported that Nrf2 exerts anti-inflammatory and anti-oxidation effects through initiating the transcription of a range of downstream genes, mainly including NQO1 and HO-1 [36]. HO-1 participates in a critical protective mechanism that is activated during cellular stress [37] and is regarded as an adaptive cellular response against inflammatory responses and oxidative injury [38]. In EAN, HO-1 is predominantly expressed starting 11 days after immunization, with local expression persisting long after neurologic signs disappear [39]. Consistent with the findings from the previous study, we observed that HO-1 expression is detectable during the peak phase of spontaneous EAN. Furthermore, in DMF-treated EAN rats, both the production of HO-1 and Nrf2 were much greater than that in the control group. Considering HO-1 induction can affect macrophage polarization toward the M2 phenotype in vivo and ex vivo [22, 40], it is reasonable to hypothesize that DMF induces macrophages to polarize toward the M2 type by upregulating the level of HO-1 and Nrf2. Therefore, we further explored the expression of Nrf2 in macrophages, and result showed that treating EAN rats with DMF can upregulate its expression in macrophages. Besides, it was proposed that in mice and humans, mechanisms underlying DMF’s protective effects in inflammatory autoimmune diseases also involved the inhibition of Th1/Th17 cells and induction of Th2 cells [41]. The authors attributed the polarization of T cells to the induction of HO-1 and thus the increased level of type II dendritic cells [41]. Therefore, the effects seen in present study on macrophages in vivo could also be secondary due to dendritic cell-mediated immune regulation. Additional in vitro investigations are needed to confirm the effects of DMF on the expression of HO-1 in macrophages and to clarify the role of HO-1 and dendritic cells in the polarization of macrophages in EAN.

DMF has been demonstrated to reduce inflammation in autoimmune and inflammatory animal models [23, 24, 33, 41–44]. In accordance with previous reports, the present study showed that DMF reduced the inflammatory response in EAN rats and favored the outcomes. DMF reduced the mRNA expression of IFN-γ, TNF-α, IL-6, and IL-17 and upregulated the mRNA expression of IL-4 and IL-10 in the spleens of EAN rats. In addition, the same trend in cytokine profile was observed in the ELISA analysis of the supernatants of cultured spleen MNCs. These data revealed that the improvement of inflammatory environment by DMF was a basis for the protective effect in EAN. Although these data were obtained from spleen samples and may not exactly represent changes in peripheral nerves in situ, as they were obtained from animals after in vivo administration of DMF, they may nonetheless provide mechanistic insight into the physiological activities underpinning this potentially valuable therapeutic and shed light on the positive clinical outcome we observed.

## Conclusions

We have demonstrated that DMF promotes EAN amelioration through a Nrf2/HO-1-mediated phenotypic shift in macrophages. The anti-inflammatory effects of DMF also improve the environmental milieu in immune organ and indirectly exert neuroprotective effects.

## Additional file

Additional file 1: Figure S1. DMF promotes M2 macrophage polarization in EAN. DMF was administered orally to EAN rats (*n* = 6 in each group) from day 1 to day 16 or day 7 to day 16 post-immunization for preventative or therapeutic treatment, respectively. On day 16 post-immunization, sciatic nerves of each group of rats were harvested for fluorescence immunohistochemistry to analyze the proportion of M1 macrophages and M2 macrophages in EAN rats after DMF treatment paradigms. (A) Fluorescence photomicrographs showing M1 and M2 macrophages in the sciatic nerves of EAN rats. Tissue sections were immunofluorescence stained for markers iNOS of M1, Arg1 of M2 macrophages, and Iba1 of general macrophages as indicated. Scale bar is 50 μm. (B) Quantitation of immunohistochemistry. Counts per square millimeter of Iba1^+^/iNOS^+^ cells (M1 phenotype) in the sciatic nerve showed that DMF reduced the number of M1 macrophages in both DMF treatment paradigms. (C) Counts per square millimeter of Iba1^+^/Arg1^+^ cells (M2 phenotype) showed that DMF-treated groups increased the number of M2 macrophages in both DMF treatment paradigms compared to the CMC group (*p* < 0.05). (DOCX 399 kb)

## Abbreviations

AUC: area under the curve
CMAP: compound muscle action potential
CMC: carboxy methyl cellulose
DMF: dimethyl fumarate
EAN: experimental autoimmune neuritis
EMG: electromyography
GBS: Guillain–Barré syndrome
HO-1: hemoxygenase-1
LFB: luxol fast blue
MNC: mononuclear cell
MNCV: motor nerve conduction velocity
MTS: 3-(4,5-dimethylthiazol-2-yl)-5-(3-carboxymethoxyphenyl)-2-(4-sulfophenyl)-2H-tetrazolium
TNF-α: tumor necrosis factor α
Nrf2: nuclear factor erythroid-derived 2-related factor 2
PNS: peripheral nervous system
