# Supplementary material for: Dimethyl fumarate attenuates experimental autoimmune neuritis through the nuclear factor erythroid-derived 2-related factor 2/hemoxygenase-1 pathway by altering the balance of M1/M2 macrophages
Source: J Neuroinflammation. 2016 May 3;13:97. doi: 10.1186/s12974-016-0559-x (PMC4855950; doi:10.1186/s12974-016-0559-x)

**Supplemental material**

**Supplemental Figure Legends**

**Supplemental Figure 1. DMF promotes M2 macrophages polarization in EAN**

DMF was administered orally to EAN rats (n=6 in each group) from day 1 to day 16 or day 7 to day 16 post-immunization for preventative or therapeutic treatment, respectively. On day 16 post-immunization, sciatic nerves of each group of rats were harvested for fluorescent immunohistochemistry to analyze the proportion of M1 macrophages and M2 macrophages in EAN rats after DMF treatment paradigms. (A) Fluorescence photomicrographs showing M1 and M2 macrophages in the sciatic nerves of EAN rats. Tissue sections were immunofluorescence stained for markers iNOS of M1, Arg1 of M2 macrophages and Iba1 of general macrophages as indicated. Scale bar is 50 μm. (B) Quantitation of immunohistochemistry. Counts per mm^2^ of Iba1^+^/iNOS^+^ cells (M1 phenotype) in sciatic nerve showed that DMF reduced the number of M1 macrophages in both DMF treatment paradigms. (C) Counts per mm^2^ of Iba1^+^/Arg1^+^ cells (M2 phenotype) showed that DMF-treated groups increased the number of M2 macrophages in both DMF treatment paradigms compared to the CMC group (p < 0.05).

**Supplemental Figure 1.**


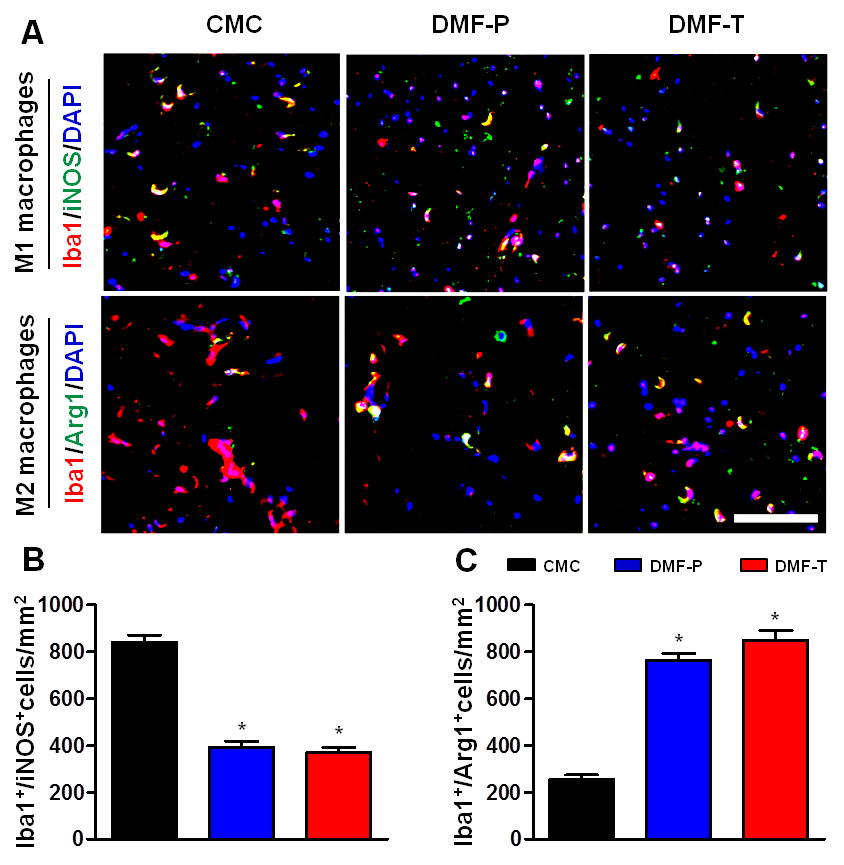

Supplement: Additional file 1: Figure S1. — DMF promotes M2 macrophage polarization in EAN. DMF was administered orally to EAN rats (n = 6 in each group) from day 1 to day 16 or day 7 to day 16 post-immunization for preventative or therapeutic treatment, respectively. On day 16 post-immunization, sciatic nerves of each group of rats were harvested for fluorescence immunohistochemistry to analyze the proportion of M1 macrophages and M2 macrophages in EAN rats after DMF treatment paradigms. (A) Fluorescence photomicrographs showing M1 and M2 macrophages in the sciatic nerves of EAN rats. Tissue sections were immunofluorescence stained for markers iNOS of M1, Arg1 of M2 macrophages, and Iba1 of general macrophages as indicated. Scale bar is 50 μm. (B) Quantitation of immunohistochemistry. Counts per square millimeter of Iba1+/iNOS+ cells (M1 phenotype) in the sciatic nerve showed that DMF reduced the number of M1 macrophages in both DMF treatment paradigms. (C) Counts per square millimeter of Iba1+/Arg1+ cells (M2 phenotype) showed that DMF-treated groups increased the number of M2 macrophages in both DMF treatment paradigms compared to the CMC group (p < 0.05). (DOCX 399 kb) [file 12974_2016_559_MOESM1_ESM.docx]
